# An Innovative, Non-surgical, and Minimally Invasive Treatment for Peri-Implantitis: A Case Report

**DOI:** 10.7759/cureus.89356

**Published:** 2025-08-04

**Authors:** Marwan El Mobadder

**Affiliations:** 1 Laser Laboratory, Oral Surgery Department, Wroclaw Medical University, Wroclaw, POL

**Keywords:** dental implants, dentistry, disinfection, implantology, implant surface disinfection, laser dentistry, peri-implant health, peri-implantitis

## Abstract

Peri-implantitis (PI) is a progressive inflammatory condition characterized by the destruction of peri-implant tissues and alveolar bone loss around dental implants. Despite its rising prevalence, there are still no effective treatment protocols for the condition. This case report presents a relatively novel non-surgical approach for managing PI. The case involves a 57-year-old male smoker with PI around a mandibular first molar implant. Before treatment, a probing pocket depth (PPD) of 8 mm was recorded on the mesial side and 5.5 mm on the distal side using a periodontal probe (PCP UNC 15, HuFriedy, Chicago, IL). Bleeding on probing (BOP) was positive in 66.7% of all sites, and a plaque index of 2 was noted according to the Silness and Loe score. Marginal bone loss was also observed, with 2.5 threads (spiral grooves that anchor the implant in bone) exposed on the mesial and 1.5 threads on the distal. The patient underwent non-surgical treatment comprising ultrasonic mechanical debridement combined with adjunctive 1064 nm diode laser therapy. After 14 months, 4- and 3-mm PPDs on the mesial and distal aspects, respectively, were noted. Bone regeneration could not be confirmed or suspected, as no histological studies or CBCT were performed. This report demonstrates the potential efficacy of laser-assisted non-surgical protocol for managing PI. Further research with larger cohorts and longer follow-up is necessary to validate this approach as a standardized treatment option.

## Introduction

A dental implant placed in the alveolar bone, along with the surrounding peri-implant mucosa, abutment, and prosthetic crown, requires consistent maintenance and proper oral hygiene. In the absence of adequate care, the site becomes vulnerable to peri-implantitis (PI), a severe and progressive inflammatory condition [1,2]. In recent years, there has been a concerning trend among clinicians to overutilize dental implants as replacement therapy, often at the expense of treating underlying periodontitis. This approach has contributed to a growing number of complications over time, notably PI, for which predictable and effective treatment options remain limited and are often unsatisfactory [3]. The European Federation of Periodontology (EFP), during its 7th workshop, defined PI as a chronic inflammatory process with bleeding on probing and marginal bone levels reduced by more than 2 mm from baseline post-bone remodeling, along with probing depths indicating pocket formation [4].

In terms of treatment of PI, Lang et al. [5] proposed the cumulative interceptive supportive therapy (CIST) protocol, comprising conservative measures like mechanical debridement and oral hygiene for shallow pockets (<3 mm) [5]. However, no universally accepted treatment protocol for PI has been established to date. Hence, there is an urgent need for effective protocols to treat and/or prevent PI. Among these approaches, different laser wavelengths and approaches seem to offer promising solutions [5]. Nonetheless, the American Academy of Periodontology (AAP) concluded that laser therapy, when used in combination with non-surgical treatment, appears to offer minimal short-term benefits in terms of probing depth reduction, clinical attachment gain, plaque reduction, and bleeding on probing. The focus was primarily on Er: YAG, CO₂, and diode lasers [6]. The AAP called for further research to strengthen the available evidence [6]. Among the different laser wavelengths, the 1064 nm laser demonstrates effective biofilm reduction primarily through a photothermal mechanism. Its energy is absorbed by titanium surfaces and biological tissues, causing a localized temperature increase that breaks the chemical bonds between the biofilm and implant surfaces without damaging the titanium. Additionally, this wavelength allows for deep tissue penetration, bacterial toxin deactivation, and protein denaturation, enhancing its overall antibacterial efficacy. This case report illustrates the management of PI using a novel 1064 nm diode laser-assisted protocol. It involved a one-year follow-up.

## Case presentation

A 57-year-old male patient with no systemic disease that could affect his periodontal and peri-implant health was referred to the clinic with complaints of a chronic sensation of pressure and discomfort on his posterior right mandibular arch. The patient was a heavy smoker (more than 10 cigarettes a day) and non-diabetic. He had no history of periodontitis. The patient mentioned that he used to visit his dentist for a professional scaling and root planing every six months after the placement of the implant. Additionally, proper oral hygiene instructions had been given to him by his dentist.

**Figure 1:**
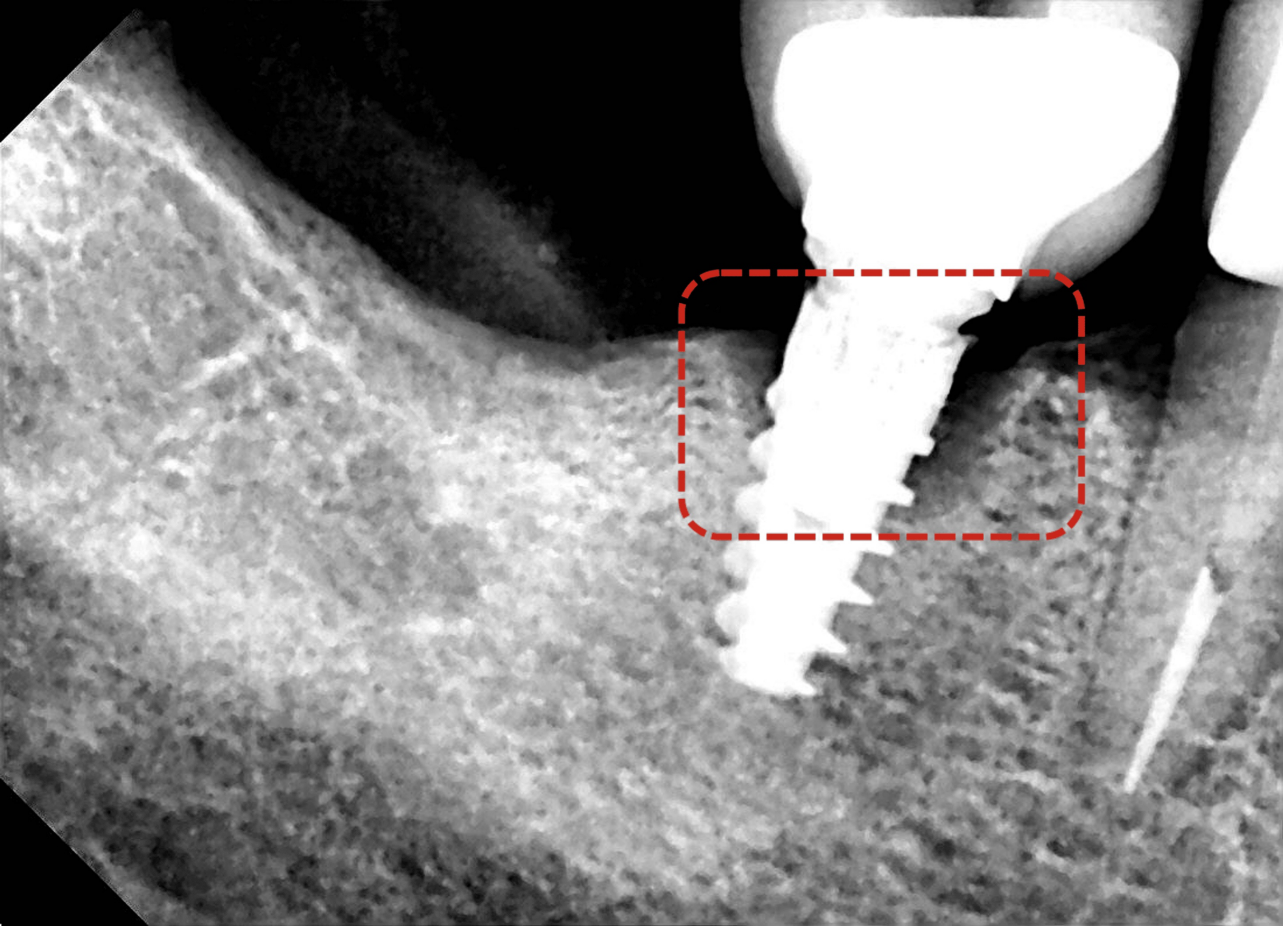
Preoperative periapical X-ray of the affected area Note the marginal bone loss around the implant, with approximately  2.5 mm of exposed threads on the mesial side and about 1.5 mm on the distal side

Clinical examination revealed inflammation around the crown of a previously placed implant in the area of the right mandibular first molar (#46). The implant had been placed on September 7, 2022, as confirmed by the dentist who performed the procedure. The clinical peri-implant parameters in the concerned area (#46) were as follows: abundant plaque on the cervical margin, positive bleeding on probing (BOP) without suppuration, a mesial probing pocket depth (PPD) of 8 mm, and a distal PPD of 5.5 mm. The measurements were made using a periodontal probe (PCP UNC 15, HuFriedy, Chicago, IL). The periapical X-ray showed a significant loss of the alveolar bone around the implant on both the mesial and the distal aspects. The bone loss was measured to be equal to ±2.5 threads on the mesial aspect and ±1.5 threads on the distal aspect. Moreover, no vertical bone loss and no PPD >5 mm were noted in other areas of his oral cavity. A diagnosis of PI on the implant replacing the 46 was established (Figure 1, Table 1), based on the European Federation of Periodontology and AAP's classification of periodontal and peri-implant disease.

**Table 1:** Overview of the patient’s condition before starting treatment

Parameters	Values
Sex	Male
Age, years	57
Diabetes	No
Smoking	10–15 cigarettes/day
Periodontal history	No history of periodontitis
Periodontal therapy	Scaling root planning every 6 months
Plaque index (Silness and Loe)	3
Bleeding on probing	66.7%
Mesial probing pocket depth #46	8 mm
Distal probing pocket depth #46	5.5 mm
Gingival recession mesial and distal #46	0 mm
Distal marginal bone loss	±1.5 threads
Mesial marginal bone loss	±2.5 threads

Treatment protocol

After obtaining the patient’s written informed consent and explaining the procedure, a non-surgical protocol for the management of PI was suggested. The protocol consisted of a mechanical debridement and a laser-assisted disinfection. A local anesthesia with articaine hydrochloride (4%) (Septanest with 1:100000 adrenaline, Septodont, Saint-Maur-des-Fossés, France) was administered in the affected area. After 10 minutes, non-surgical mechanical debridement was performed using ultrasonic instrumentation with S and S1 inserts without touching the implant surface and IP1 ultrasonic tip dedicated for the titanium implant surface (IP1, Newtron, Acteon, Mérignac, France) for the implant surface. Irrigation with 0.12% chlorhexidine was done during the mechanical debridement. Then, a laser-assisted approach was initiated. The laser was a 1064 nm diode laser (Smart M, Lasotronix, Warsaw, Poland) used inside the peri-implant pocket, in a contact mode with the implant surface and a pulsed mode.

**Figure 2:**
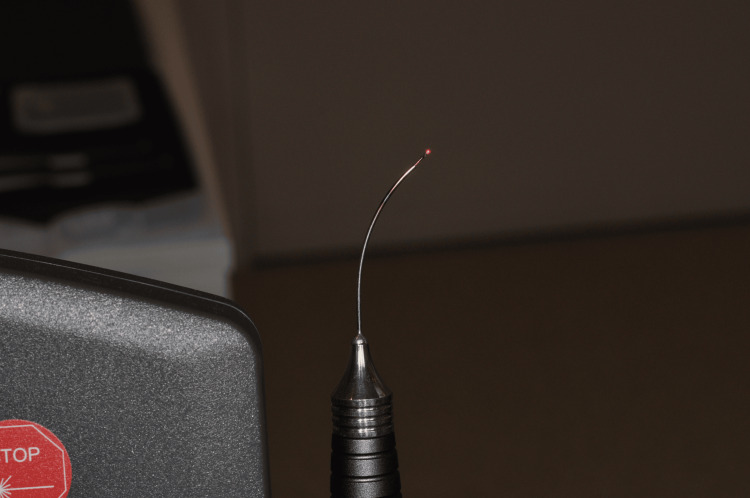
Fiber delivery system of the 1064 nm Lasotronix Smart M Laser used for the procedure

The parameters of irradiation were as follows: 1064 nm wavelength of a diode laser in a pulsed and contact mode (Smart M, Lasotronix, Warsaw, Poland), 10.0 W peak power, 3 ms pulse duration (TON), a pause time of 100 ms (TOFF), frequency of 9.71Hz, duty cycle of 2.91%, energy per pulse of 30 mJ and an average power of 291 mW. The fiber diameter of the laser was 320 microns (Lasotronix laser fiber, Warsaw, Poland). The irradiation was made for 4 seconds. The irradiation was repeated in four areas around the affected implant (vestibular, mesial, distal, and lingual) with exactly the same parameters. The parameters were chosen based on a thorough understanding of laser-tissue interactions and safety. The aim was to achieve decontamination without causing any harm or damage to the implant surface. The treatment was done on March 11, 2024 (18 months after implant placement). No local or systemic antibiotics were prescribed to the patient. He was prescribed a mouthwash containing 0.12% chlorhexidine to be used twice daily for 45 seconds over a period of 10 days. In addition, maintenance sessions every six months were recommended. Figure 2 shows the fiber delivery system of the 1064 nm Lasotronix Smart M Laser used for the procedure.

Follow-up

**Figure 3:**
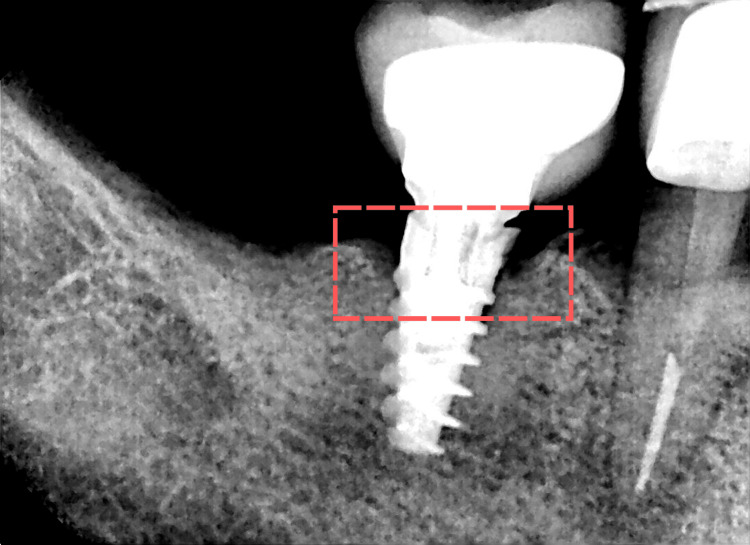
Postoperative periapical X-ray of the affected area at 14 months Note the marginal bone around the implant, showing an overall improvement in bone level, which may indicate possible healing. However, this could not be confirmed, as no CBCT or histological analysis was performed

A follow-up was conducted after 14 months, during which a complete healing of the peri-implant soft tissue was noted with no signs of inflammation. There was no bleeding on probing, and the plaque index was 1. The peri-implant pocket depth was equal to 4 mm on the mesial and 3 mm on the distal, indicating the resolution of the inflammatory process. The patient reported no sensation of pressure or discomfort. Moreover, he experienced no complications or side effects and stated that he was satisfied with the results. Parallel radiographic examination revealed notable improvement in the marginal bone level, approximately ±1 thread on the mesial aspect and ±1.5 threads on the distal aspect. However, since neither a CBCT nor a histological study was performed, bone healing could not be confirmed (Figure 3).

## Discussion

The rising prevalence of dental implants, often placed without sufficient attention to risk factors such as residual periodontal disease, smoking, or inadequate maintenance, inevitably leads to a rise in peri-implant complications [1]. PI remains a major challenge in dentistry due to its relatively rapid progression, significant local and systemic impact, and the limited and unpredictable efficacy of current treatment modalities [6]. This clinical case employed a minimally invasive laser-assisted protocol combined with conventional non-surgical mechanical debridement, using a 1064 nm diode laser for disinfection. The treatment resulted in significant improvement in peri-implant mucosal health, radiographic signs of condition enhancement, and stabilization of the peri-implant tissues over 14 months, as evidenced by probing depth reduction and decreased BOP. However, since no three-dimensional radiographic evaluation, such as CBCT, was performed and no histological analysis was conducted, bone regeneration cannot be definitively confirmed. Future studies incorporating these advanced imaging techniques and histological assessments are essential to accurately verify the extent of bone healing.

This report underscores the potential of adjunctive laser therapy as a conservative alternative to immediate surgical intervention in PI management. The properties behind the effectiveness of the 1064 nm diode laser primarily involve its photo-thermal action. The laser energy, absorbed by the titanium implant and surrounding tissues, induces a localized temperature rise that disrupts biofilm adhesion via thermal-photo-desorption, effectively decontaminating the implant surface without damaging it [7]. The near-infrared wavelength allows deep tissue penetration, bacterial toxin deactivation, protein denaturation, and coagulation, which collectively enhance the antimicrobial effect [7].

Current literature supports this approach; for example, a systematic review by the AAP concluded that lasers, when used adjunctively with surgical or non-surgical therapy, provide modest benefits in reducing probing depth and BOP, particularly in non-surgical settings [6]. However, the overall clinical improvements remain limited, emphasizing the importance of combining laser therapy with mechanical debridement rather than relying on laser monotherapy. Moreover, our study’s findings align with those of a study by Chen et al., where Er: YAG laser irradiation led to significant probing depth reduction and biofilm disruption, complementing mechanical debridement. It is important to emphasize that different wavelengths have varying effects on tissues [8]. In our case, the 1064 nm wavelength showed a positive impact, whereas in Chen et al.'s study, the Er: YAG wavelength also demonstrated beneficial effects. Therefore, future studies should focus on comparing the effectiveness of different wavelengths [8].

Furthermore, laser-assisted regenerative surgical therapy studies, such as a study by Wang et al., demonstrated that adjunctive Er: YAG laser use during regenerative surgery yielded greater probing depth reductions compared to conventional treatment alone, although clinical attachment level (CAL) gains and radiographic bone fill differences were not statistically significant [9]. This suggests that while laser application may enhance soft tissue healing and inflammation control, hard tissue regeneration likely depends on multiple factors, including defect morphology and patient compliance [9].

The favorable outcomes in our case highlight the importance of individualized treatment planning. While non-surgical laser-assisted therapy may be sufficient for moderate peri-implant inflammation without suppuration, surgical intervention remains necessary in advanced cases with granulation tissue and pus formation [10]. This nuanced approach aligns with the reviewed evidence that laser adjuncts improve clinical parameters modestly but do not replace the need for comprehensive treatment [10]. Limitations of this report include its single-case design and the relatively short follow-up period, which, while adequate for short-term evaluation, cannot predict long-term stability. Larger randomized controlled trials with longer follow-ups are necessary to confirm the adjunctive benefits of 1064 nm diode laser therapy in PI treatment. Despite these limitations, this report adds to the growing evidence supporting conservative, biologically driven management of PI. Adjunctive use of the 1064 nm diode laser appears to enhance non-surgical therapy outcomes by improving mucosal health and supporting bone repair, underscoring the value of integrating laser technology in routine clinical practice.

## Conclusions

This report highlights the potential of minimally invasive laser-assisted therapy as an adjunct to conventional mechanical debridement in the non-surgical management of PI. The clinical and radiographic improvements observed, particularly in probing depth, BOP, and marginal bone stability, suggest that this approach may offer promising short-term benefits in selected cases. However, as this report only involves a single case, the findings are preliminary and cannot be generalized. Definitive conclusions regarding bone regeneration or long-term stability cannot be drawn in the absence of three-dimensional imaging or histological validation. Controlled clinical trials with larger sample sizes and long-term follow-up are necessary to confirm the efficacy, predictability, and reproducibility of this treatment approach. Nonetheless, this report contributes valuable clinical insights, encouraging clinicians to consider non-surgical alternatives before proceeding to more invasive procedures, particularly in cases where early intervention may preserve implant function.
